# SARS-CoV-2, Cardiovascular Diseases, and Noncoding RNAs: A Connected Triad

**DOI:** 10.3390/ijms222212243

**Published:** 2021-11-12

**Authors:** Lucia Natarelli, Fabio Virgili, Christian Weber

**Affiliations:** 1 Institute for Cardiovascular Prevention (IPEK), Ludwig-Maximilians-Universität (LMU), 800336 Munich, Germany; christian.weber@med.uni-muenchen.de; 2 Research Center for Food and Nutrition, Council for Agricultural Research and Economics, 00178 Rome, Italy; fabio.virgili@crea.gov.it; 3 German Center for Cardiovascular Research (DZHK), Partner Site Munich Heart Alliance, 80336 Munich, Germany; 4 Department of Biochemistry, Cardiovascular Research Institute Maastricht (CARIM), Maastricht University, 6229 HX Maastricht, The Netherlands; 5 Munich Cluster for Systems Neurology (SyNergy), Institute for Stroke and Dementia Research, 81377 Munich, Germany

**Keywords:** SARS-CoV-2, COVID-19, cardiovascular diseases, noncoding RNA

## Abstract

Coronavirus Disease 2019 (COVID-19), caused by the Severe Acute Respiratory Syndrome Coronavirus 2 (SARS-CoV-2), is characterized by important respiratory impairments frequently associated with severe cardiovascular damages. Moreover, patients with pre-existing comorbidity for cardiovascular diseases (CVD) often present a dramatic increase in inflammatory cytokines release, which increases the severity and adverse outcomes of the infection and, finally, mortality risk. Despite this evident association at the clinical level, the mechanisms linking CVD and COVID-19 are still blurry and unresolved. Noncoding RNAs (ncRNAs) are functional RNA molecules transcribed from DNA but usually not translated into proteins. They play an important role in the regulation of gene expression, either in relatively stable conditions or as a response to different stimuli, including viral infection, and are therefore considered a possible important target in the design of specific drugs. In this review, we introduce known associations and interactions between COVID-19 and CVD, discussing the role of ncRNAs within SARS-CoV-2 infection from the perspective of the development of efficient pharmacological tools to treat COVID-19 patients and taking into account the equally dramatic associated consequences, such as those affecting the cardiovascular system.

## 1. Introduction

Detected in Wuhan City, China, and rapidly spreading worldwide, SARS-CoV-2 has been identified as the causative agent of Coronavirus Disease 2019 (COVID-19), defined as a pandemic in 2020 by the World Health Organization (WHO). One of the suggested reasons for the extremely high SARS-CoV-2 infectivity resides in the high mutation rate of the viral genome, intrinsic of viral adaptation. In fact, several studies reported that SARS-CoV-2, different from other RNA-based viruses, presents an exceptionally high mutation rate. The most effective mutations involve the protein-coding genome regions [1,2,3,4], in particular, the sequence encoding the Spike protein [5], which plays a key role in receptor recognition and the host–cell membrane fusion process, thus generating a beneficial trait for virus adaptation [1,2]. Obviously, mutations also occur in noncoding regions of the viral genome, such as those reported to occur at the 3′ end of the SARS-CoV-2 genome, while no data dealing with the effects of mutations at 3′ mutation are available [1,2]. Interestingly, the 5′ end and the leader sequence of SARS-CoV-2 are apparently neither affected by evident mutations nor correlated with the severity of COVID-19 disease [1,2]. The presence of mutations in the leader sequence may collide with coronavirus surveillance since the leader sequence is highly conserved within all SARS-CoV and SARS-CoV-2 variants and is crucial for viral replication. This observation may drive researchers to consider the 5′ end and the leader sequence as candidate targets while designing novel antiviral drugs. Hence, although the efficacy of RNA-based novel vaccines is higher than that of previous vaccines, the high mutation rate reported in the Spike protein suggests that therapies based on Spike protein may be ineffective in a long-term perspective.

In addition to respiratory impairments, a number of patients experienced severe cardiovascular damages [6], and those with comorbidities, such as cardiovascular diseases (CVD), were challenged by a further dramatic increase in inflammatory cytokine release, the severity and adverse outcomes of Acute respiratory distress syndrome (ARDS), and finally mortality risk [6]. Although the mechanisms underlying this association are still uncertain, the presence of pre-existing CVD in patients infected by COVID-19 is of paramount importance in defining proper therapies and a timely treatment of patients to reduce mortality. Several studies demonstrated the existence of potential noncoding RNAs (ncRNAs), such as micro- and long noncoding RNAs (miRNAs, lncRNAs), suitable for binding and inhibiting the viral genome during viral infections as well as for potentially titrating on viral genomes and therefore redirecting from their canonical targets. Notably, the roles of ncRNAs in CVD, myocardial injury, and inflammation are well-documented, but their roles as a functional link between CVD and COVID-19 disease are still unknown. However, current drugs based on ncRNAs seem to be capable of targeting both the Spike protein transcript and low mutation regions of the SARS-CoV-2 genome.

Herein, we review the current findings and hypothesis on molecular mechanisms linking SARS-CoV-2 and CVD. When addressing the therapeutic interventions against SARS-CoV-2 infection and CVD, we specifically focus on current and alternative RNA-based antiviral strategies, including those utilizing micro- and long noncoding RNAs (miRNAs and lncRNAs).

## 2. SARS-CoV-2 Genome

Similar to all members of the coronaviruses family, SARS-CoV-2 is an enveloped virus with a positive single-strand RNA genome showing an 82% sequence identity with SARS-CoV and a 50% with MERS-CoV [7]. Its genome is about 30 kb in length with a 5′ cap and a 3′ polyadenilated end, and it is composed of several open reading frames (ORF1ab, ORF3a, ORF6, ORF7a, ORF7b, ORF8, and ORF10) located in the 3′ region encoding for the replicase, membrane, envelope, Spike glycoprotein, and nucleocapsid proteins and for the accessory and nonstructural proteins. SARS-CoV-2 contains a highly conserved sequence of 90 nucleotides (nt) at the 5′ end termed “leader sequence”, followed by a cis-acting transcription-regulating sequence (TRS), pivotal for viral mRNA transcription [8,9]. 

As mentioned above, the Spike protein plays a fundamental role in SARS-CoV-2 infectivity. It is a trimeric protein containing an N-terminal domain, a surface subunit (S1), and a transmembrane subunit (S2). The S1/S2 junction is usually cleaved by the host cell protein protease Furin [10] to generate the Spike active form. S1 includes the signal sequence, the N-terminal domain, a receptor-binding domain, and the neuropilin-1 binding domain. S2 contains heptad repeat domains, the fusion peptide, a transmembrane domain, and a short cytoplasmic end. 

The mechanism of infection by SARS-CoV-2 relies on the binding of the Spike protein to the enzymatic domain of the angiotensin-converting enzyme 2 (ACE2) receptor exposed on the surface of several cell types, including alveolar, epithelial, and endothelial cells; cardiac myocytes; monocyte/macrophages; and neurons [11]. The binding between Spike and ACE2 promotes either the endocytic transfer of SARS-CoV-2 into endosomes [12] or the direct fusion of the viral envelope with the host cell membrane [13]. The binding affinity between Spike and ACE2 is at least in part dependent on electrostatic—opposite—charges. Although the overall total charge of SARS-CoV-2 is positive due to positively charged structural proteins, Spike presents an imbalance for a positive charge that is likely to increase its affinity with ACE2 and to facilitate infection of the ACE2-presenting cells. This hypothesis has been proposed to explain the high virulence of SARS-CoV-2 in comparison with other viruses belonging to the same family [14]. 

## 3. The Relationship between SARS Infections and CVD 

### 3.1. Diseases Associated with Viral Infection

The first clinical manifestations of pneumonia cases were reported in Wuhan, China, on 2 January 2020, when 41 patients admitted to hospitals with a laboratory-confirmed 2019-CoV infection showed common symptoms of fever, dyspnea, cough, myalgia or fatigue, and pneumonia [6]. Nausea and diarrhea were considered atypical symptoms. However, during hospitalization, patients who were infected underwent increased coagulation activation, cellular immune deficiency, and myocardial injury [6]. Twelve percent of the patients progressed toward cardiac injury, including acute myocardial infarction (MI), mainly manifested by elevated high-sensitivity cardiac troponin I levels [6]. Right after the assignment as a “pandemic” by the WHO, Guo et al. associated COVID-19 disease with MI more consistently in March 2020, reporting that 35.3% of 187 patients who were hospitalized had underlying CVD, including hypertension, coronary artery disease (CAD), and cardiomyopathy. Out of this group, 27.8% evolved a myocardial injury [15]. Patients with CVD exhibited an increased mortality rate (13.3%), and the percentage dramatically increased in patients with CVD together with high troponin I levels (69.4%) [15]. Taken together, several cohort studies demonstrated that pre-existing CVD in patients infected by COVID-19 was significantly associated with an increased severity of ARDS and adverse outcomes. Patients with comorbidities were characterized by elevated troponin levels, inflammatory markers, and frequent malignant arrhythmias (71%) [6,16,17]. Two of the main parameters detected in patients with an uncontrolled SARS-CoV-2 infection were increases in pro-inflammatory cytokines and chemokines, such as IL-1β, IL-6, and tumor necrosis factor-α [18], paralleled by high coagulation markers, eventually leading to thromboembolic events [19,20,21]. Even though inflammation and thrombogenic events were strongly associated with CVD, the mechanisms underlying the interaction between SARS-CoV-2 and CVD were and still apparently are largely unknown.

In addition to respiratory impairments, patients infected with the 2019-nCoV virus in Wuhan developed severe cardiovascular damage [6]. Those with comorbidities, such as CVD, had a further increase in inflammatory cytokines and an increased risk of mortality [6,17]. Additional meta-analysis reports [22,23] suggested a cumulative contribution from different metabolic disorders such as hypertension, diabetes mellitus (DM), obesity, CAD, and CVD to SARS-CoV-2 infection, COVID-19 severity, and mortality [17,23]. In the details, Zhou et al. [17] reported observations from 54 subjects who died during hospitalization out of a group of 191 patients. Forty-eight percent of patients who died had a comorbidity, with hypertension being the most common (30%), followed by DM (19%) and CAD (8%), or a combination of these pathologies. Similarly, the National Health Commission of China (NHC), reported that 35% of patients infected by and hospitalized with COVID-19 were affected by hypertension and that 17% had CAD [24]. 

Similar to COVID-19, a clinical correlation between MERS-CoV infection and acute myocarditis has been reported by Alhogmani, who described a patient with dyspnoea, chest pain, and arrhythmias, typical of acute myocarditis [25]. This patient, positive for MERS-CoV, showed evidence of myocardial injury; elevated troponin levels; and negative viral infection levels for all viruses known to cause myocarditis, such as influenza, adenovirus, hepatitis C, cytomegalovirus, and parvovirus B19. Patients infected by SARS-CoV-2 present a range of myocardial damages similar to those reported for patients infected by MERS-CoV [6], but their treatment is apparently more difficult [6]. According to this complexity, together with canonical adopted therapies, such as steroids, and antiviral and intravenous immunoglobulins, patients infected by COVID-19 were co-treated with glucocorticoids. Noteworthily, the impact of glucocorticoids is unclear, and therefore, this treatment protocol is no longer recommended by the WHO unless patients present a chronic obstructive pulmonary disease (https://www.who.int/publications/i/item/WHO-2019-nCoV-clinical-2021-1 (accessed on 5 October 2021)).

Most patients diagnosed with COVID-19 reported by the NHC [24] presented chest tightness and heart palpitation rather than respiratory symptoms. Among those who died, 11.8% of total patients without underlying CVD preceding the infection had evident heart injury, showing elevated high-sensitivity cardiac troponin I levels or an occurring cardiac arrest when hospitalized [26]. 

Although initial observations suggested a correlation between age (>50 years old) and the chance to develop cardiovascular complications, a study conducted in the United Kingdom reported about four out of a total of eight children positive for SARS-CoV-2 presenting symptoms corresponding to Kawasaki disease, including a hyper inflammatory shock, coronary vessel abnormalities, and cardiac dysfunction [27]. However, a large number of following reports did not unequivocally confirm whether SARS-CoV-2 infection promotes systemic inflammation in cardiac cells and myocarditis or whether myocarditis was preceding viral infection. Mechanistically, it is plausible that SARS-CoV-2, similarly to SARS- and MERS-CoV, directly infects host cells through the ACE2 receptor, highly expressed from cardiac cells [24,28]. Accordingly, a gene expression analysis demonstrated that human ventricular myocardium cells from patients infected by SARS-CoV-2 express high levels of ACE2 and furin, both indispensable for viral infection [29].

### 3.2. Increased Susceptibility to Negative Outcomes Due to Pre-Existing Pathologies

Comorbidity emerged as factor increasing the risk of infection by SARS-CoV-2, especially in patients with CVD, CAD, DM, and hypertension. Indeed, patients with pre-existing CVD and infected with SARS-CoV-2 showed increased severity of ARDS, cardiac insufficiency, and high mortality rate. A family cluster study from Chan et al. indicated that patients severely affected by SARS-CoV-2 had pre-existing hypertension, heart disease, arrhythmia, and age-associated severe pneumonia [30]. Although the link between cardiovascular system and viral pathology is unknown, it has been proposed that viral pathology might generate more severe complications in patients with chronic CVD due to an imbalance between CVD-related reduced cardiac function and infection-induced increase in metabolic demand [31]. Moreover, patients with CAD are likely to be more susceptible to viral infection due to plaque rupture, secondary to virally induced systemic inflammation, suggesting plaque-stabilizing therapies as an expedient therapeutic strategy [31]. As mentioned above, the mechanism of infection by SARS-CoV-2 is based on viral Spike recognition and infection of cells expressing the ACE2 receptor. ACE2 is also expressed in the cardiovascular system, and it has been demonstrated that SARS-CoV can downregulate ACE2-related pulmonary and myocardial pathways promoting lung and myocardial inflammation [24,32]. These evidence provide an additional link between the cardiovascular system and SARS-CoV-2 infection.

Overall, the increased systemic inflammation in patients infected by COVID-19 and the relation with cardiovascular damages underline the priority of developing potential therapies to treat patients infected by COVID-19 with cardiovascular co-morbidities.

## 4. Noncoding RNAs

Whole genome and RNA-seq-based studies revealed that only 2% of the genome codifies for protein-coding RNAs, whereas almost 70% of the transcripts are not translated into a protein and are therefore referred as noncoding RNAs (ncRNAs). NcRNAs are further divided in 10 additional RNA subclasses, which include RNA transfers (tRNA), ribosomal RNAs (rRNA), small nuclear RNAs (snRNA), piwi-interactin RNA clusters (piwi RNA), microRNAs (miRNAs), and long noncoding RNAs (lncRNAs). Together with novel circular RNAs (circRNAs), ncRNAs play pivotal roles in the onset and progression of several diseases, including cardiovascular and metabolic diseases, and in general during inflammation [33]. 

According to the HUGO Gene Nomenclature Committee (HGNC, http://www.genenames.org (accessed on 5 October 2021)), the term miRNA refers to molecules 21–25 nt in length. They regulate gene expression by enhancing RNA degradation and translational repression of their target transcript in the RNA-induced silencing complex (RISC). Not only protein-coding RNAs but also lncRNAs and proteins can be targets of miRNAs [34,35]. MiRNAs bind to their RNA targets through 2–7 nt located at the 5′ end of their sequence, named the seed sequence, to induce degradation or translational inhibition of their targets [36,37]. Since RNAs can share the same target site for different miRNAs, and conversely, a single miRNA can modulate the expression of several RNAs; this plasticity is likely to affect a very large spectrum of diverse biological processes [36].

LncRNAs are >200 nt in length and comprise a more heterogeneous class of RNAs. Mature and functional lncRNAs, transcribed and spliced by RNApol II and III, can be derived from enhancer or promoter regions, intronic regions of protein-coding RNAs, antisense strands, lncRNA genes, or intergenic regions (lincRNAs). Circular RNAs (circRNAs) are also members of the lncRNA subclass and are derived from back splicing of exons [38]. Being usually packaged in exosomes, where they can bind miRNAs, circ- and lncRNAs are considered representative biomarkers in several pathologies. They show a broad spectrum of activities, acting as epigenetic regulators of gene expression and as transcriptional and translational modulators of mRNA splicing, or can interact with proteins. They are miRNA sponges or can be targets of miRNAs. Moreover, circRNAs can be released in the circulation and therefore dysregulated, such as lncRNAs, in several diseases [39,40].

Even though a detailed description of ncRNA synthesis and delivery is out of the scope of this review, it is worth mentioning that ncRNA can interact with viral genomes either inside the expressing cells, and in particular, within target cells, after being delivered to in the blood vessels. Circulating ncRNAs may originate either from immune cells or from endothelial cells of other organs. In the blood stream, miRNAs circulate as microparticles cargo (exosomes, microvesicles, and apoptotic body) or are associated with specific RNA-bind proteins, such as Argonaute-2 (Ago2), and with lipoproteins [41].

### 4.1. NcRNAs in Viral and SARS-CoV-2 Infection

As mentioned, ncRNAs are master regulators of cell functions. In particular, miRNAs and lncRNAs are involved in the interplay between viruses and host cells upon infection. Indeed, most viral genomes contain miRNA response elements (MREs) at their 5′ and 3′ ends that support miRNA-mediated antiviral response [42,43,44]. Viral mutation can be therefore considered a consequential “response” allowing for the avoidance of recognition by host miRNAs [42,43,44]. Several studies demonstrated the existence of potential miRNAs suitable to bind and inhibit viral infections by targeting the viral untranslated regions. For example, miR-1 [45], miR-222 [46,47], miR-21 [48,49], miR-449a [50], and the miR-34 family [51]. Hence, the idea of designing RNA-based drugs mimicking or inhibiting miRNA activity with a potential efficacy against RNA-based viruses is promising and is being explored. Waiting for proof-of-principle studies and clinical trials, several reports have already indicated that human host miRNAs, part of them modulated in humans upon SARS-CoV-2 infection, can potentially interact with the SARS-CoV-2 genome and transcripts [1,2,52,53,54,55,56]. For instance, miR-3661 is directly involved in the formation of SARS-CoV-2 proteins in the lungs, and similarly, miR-17-5p and miR-20b-5p are downregulated in the serum of patients infected by COVID-19 [57]. Human miR-190a-5p and miR-184a-3p have been reported to target SARS-COV-2 mRNA encoding for the ORF6 and ORF8 proteins, respectively, supporting the immune mechanism in preventing viral replication [58]. 

A comparative analysis of different SARS-CoV-2 genomes obtained by people infected worldwide identified six miRNAs, including miR-23b, miR-125a, and miR-198, that play crucial antiviral roles in respiratory diseases and contain potential binding sites in the SARS-CoV-2 genome [59]. Moreover, several miRNAs have been documented to potentially endogenously interact with ACE2, such as those belonging to the let-7 family members; miR-200b/c; miR-18, which upregulate ACE2 expression in COVID-19-associated nephropathy [60]; and miR-145, of which the overexpression downregulates the ACE protein [60]. Finally, the miR-221/222 cluster is involved in inflammatory regulation and vascular remodeling, and it has been reported to regulate ACE2 expression in COVID-19-associated nephropathy [60].

Computational approaches using available gene expression datasets from patients infected by MERS, SARS-CoV, and SARS-CoV-2 suggest a scenario in which the virus can engage host ncRNAs for its replication. Despite these data needing to be validated to determine their clinical relevance, Yousefi and colleagues identified several miRNAs engaged by SARS-CoV during host cell infection that are involved in the regulation of the TGF-beta signaling pathway, for example hsa-miR-92b, hsa-miR-23b, hsa-miR-203a, hsa-miR-125a, hsa-miR-21, let-7 family, hsa-miR-145, and hsa-miR-155 [61].

On the other hand, studies on the role of lncRNAs in viral infections are still scarce. Few lncRNAs have been reported to promote or inhibit viral replication upon infection. NEAT1 [62], EGOT [63], NRON [62], and lncRNA-CMPK2 [63] have been reported as lncRNAs regulated by the hepatitis C and B viruses (HCV and HBV) while NEAT1 is upregulated in HIV, encephalitis, and influenza infections [62,63]. 

Interestingly, recent data reported the existence of viral lncRNAs, named virus-encoded lncRNAs and chimeric lncRNAs, which can interfere with or are incorporated in the host cell genome. Among these are EBER1/2, Adenovirus (AdV) virus-associated RNA I and II (VAI, VAII RNA), and sfRNAs [64]. Adenovirus (AdV) virus-associated RNA (VA RNA) is a human essential pro-viral ncRNA known to relieve the cellular antiviral blockade of protein synthesis. Recent findings revealed that VA RNAs interfere with the Dicer-mediated miRNAs gene silencing [65]. In detail, VA RNA fragments can inhibit the export of miRNA precursors in the cytoplasm by saturating the nuclear export protein Exoprtin 5 and can saturate the ribonuclease Dicer to interfere with miRNA biogenesis. Additionally, VA RNA competing fragments can be processed by Dicer and incorporated into the RISC complex as “mivaRNAs”, thus inhibiting the mediators of the immune system [65]. Hence, viral lncRNAs may represent a novel attractive alternative in designing antiviral therapies.

Despite data on the role of lncRNAs in SARS-CoV-2 infection being scarce, computational analysis and RNA-seq data from COVID-19 patients worldwide suggest that the broad spectrum of action of lncRNAs can be a promising background upon which to design lncRNA-based vaccines to counteract SARS-CoV-2 infection [66,67]. This hypothesis is corroborated by the findings reported by Moazzam-Jazi and collaborators who identified six lncRNAs differentially modulated in peripheral blood mononuclear cells (PBMC) and broncho-alveolar lavage fluid (BALF) obtained from patients infected by COVID-19. Four out of six, namely HOTAIR, PVT1, lncRNA-PGCs, and AL392172.1, showed high affinity for the viral genome, whereas MALAT1 and NEAT1 binding may contribute to inflammatory development in infected cells [66]. Along this line, the weighted correlation network analysis from Mukherjee et al. identified four lncRNAs, WAKMAR2, EGOT, EPB41L4A-AS1 and ENSG00000271646, in which the expressions increased in SARS-CoV-2-infected cells [68]. Although their roles are partly known, WAKMAR2 and EGOT are associated with cytokine expression and might favor viral replication in the lungs [68]. In addition, a computational analysis on lncRNA interaction against the SARS-CoV-2 genome reveal that H19 can interact with the SARS-CoV-2 genome and the Spike transcript [56]. 

Similar to lncRNAs, circRNAs are implicated in the maintenance of physiological cell responses, such as immune tolerance and immune escapes. Moreover, exosomes, which play important roles in COVID-19 recurrence are relatively enriched in circRNAs. RNA-seq data from the whole blood of patients infected by COVID-19 and a pathway enrichment analysis identified 114 differentially expressed circRNAs and 10 lncRNAs in exosomes from patients infected by COVID-19 compared with healthy people [69]. Although identified circ- and lncRNA are only putative transcripts, most of them have already been reported to play a role in cardiac injury, in neuronal functions, and in lung cancer. Thus far, promising data emerged from a computational analysis of SARS-CoV-2-infected mice. Although the data need further validation in humans to determine their clinical relevance, the data from Arora et al. indicate a host–pathogen interaction based on the ceRNA network, including virus-regulated hosts lncRNA Gm26917 and circRNA Ppp1r10, acting as sponges for miR-124-3p targeting of Dtx58 [70]. In addition, similar to viral lncRNAs, recent investigations have reported the existence of SARS-CoV-2-derived circRNAs having a still unknown role and identities that differ from those encoded by human genome [71,72], supporting viral ncRNAs as novel attractive alternative candidates in designing RNA-based antiviral therapies.

Due to the high mutational range of SARS-CoV-2, future therapeutic strategies involving ncRNA-based drugs should focus on miRNAs and lncRNAs interacting with the SARS-CoV-2 3′ and 5′ ends, which show a lower mutational range in comparison with protein-coding viral transcripts [1,2]. Let-7, miR-23b, miR-21, lncRNA H19, MALAT1, and VA RNAs have already been proposed as potential candidates, and clinical trials are already in phases I or II for some of them (see Section 6).

### 4.2. Host ncRNAs in CVD and Myocardial Injury upon SARS-CoV-2 Infection

The roles of miRNAs, lncRNAs, and circRNAs in CVD, myocardial injury, DM, inflammation, and cancer are well-documented, but their role as a functional link between CVD and COVID-19 disease is still unknown. A functional analysis of genes differentially expressed in the lungs and heart after SARS-CoV-2 infection compared with in healthy patients revealed that SARS-CoV-2 increases the expression of specific miRNAs in CD8+T, CD4+T, NK cells, and CD14 cells. SARS-CoV-2 may act as a sponge of these miRNAs, being potentially titrated on the viral genome and resulting in an altered regulation of canonical miRNA targets [55]. An additional computational analysis confirmed that the SARS-CoV-2 genome potentially associates with host hsa-miR-3529-3p, hsa-miR-451b, hsa-miR-499b-3p, and hsa-miR-5688 [56,73].

Other investigations indicated miR-21 as the miRNA with the best binding to the SARS-CoV-2 genome [74]. MiR-21 regulates cardiac structure and function by modulating the ERK–MAP kinase signaling pathway in cardiac fibroblast, and it is increased during heart failure, where acts as a promoter of interstitial fibrosis and cardiac dysfunction [75]. Human miR-23b and miR-126 have also been identified as potentially able to bind SARS-CoV-2 genes [59]. Notably, miR-126-3p and miR-126-5p are known regulators of endothelial vascular biology and leukocyte adhesion [76], and their expressions are significantly affected in patients with CVD [35,77]. Recently, the work of Dongchao Lu and colleagues supported the role of miR-200c in binding and inhibiting the ACE2 transcript in rat and human iPSC-derived cardiomyocytes [78]. Since miR-200c is upregulated in CVDs, controversy still exists on the effects related to ACE2 in CVD and the role of miR-200c in COVID-19 infection.

As for miRNAs, lncRNAs and circRNAs are known to be involved in several physiological cell functions, including immune tolerance. Observational studies indicate that lncRNA H19, which promotes pulmonary arterial hypertension (PAH), promotes abdominal aortic aneurysm in mice and pigs. LncRNA LIPCAR emerged as a plasma molecular biomarker of acute myocardial infarction (AMI), since its levels increased during cardiac remodeling progression [79] concomitantly with increased levels of ACE2 proteins [80]. However, data demonstrating a direct involvement of lncRNAs and circRNAs as a molecular link between CVD, MI, and SARS-CoV-2 infection are missing. The data reported are still indirect and putative but potentially support the role of human lncRNAs as novel targets in designing antiviral therapies against viral infection in CVD patients. Among these, studies on bronchial epithelial cells and lung tissues from patients infected by SARS-CoV-2 reveal that certain host-derived lncRNAs and circRNAs, such as MALAT1, NEAT1, and HRCR, are affected in patients infected by COVID-19 [79]. Additionally, LIPCAR, H19, ANRIL, MIAT1, and SENCR were modulated in patients with CVD [79,81].

## 5. RNA-Based Drugs and Vaccines as Novel Therapeutic Approaches to Treating Patients Infected by COVID-19 

### 5.1. Canonical Therapeutic Approaches to Treating Patients Infected by COVID-19

It is important to consider that the path to the release of treatments targeting a specific infective disease is, by definition, time-consuming and therefore not a viable strategy when a pandemic such as COVID-19 arises. Thus, the strategy adopted to treat patients infected by COVID-19 was the utilization of already clinically approved antiviral and anti-inflammatory drugs. Available therapies directly act against the virus, at different stages of viral infection, or as modulators of the immune system [82]. However, they have frequently shown some cardiovascular adverse effects [83] and unwanted pharmacokinetic interactions [84,85]. In example, Lopinavir and ritonavir, originally used to treat HIV patients, chloroquine, and hydroxy-chloroquine, have been initially utilized in patients infected by COVID-19, with a certain successful rate against SARS-CoV-2. However, they interfere with cardiovascular therapies [39] and promote hypertension and arrhythmias, prolonging QT interval [31], especially when combined with other antibiotics [83,84,86].

Thus far, the utilization of monoclonal antibodies (mAB) against IL-6, such as Tocilizumab, has been considered according to evidence of a dramatic increase in proinflammatory cytokine IL6 in patients infected by COVID-19, leading to an increased generalized cytokine release and inflammation in lungs [87,88]. Preliminary data seem promising, and treatment with Tocilizumab reduces the mortality [88]. However, a number of patients infected by COVID-19 have been reported to be unaffected by this therapy, suggesting that further investigations are needed.

### 5.2. Spike: ACE2 Inhibitors, ACE2 Inhibitors, and Angiotensin Receptor Blockers

As mentioned before, SARS-CoV-2 infection of host cells involves the interaction of viral Spike with ACE2 receptor. Therefore, the inhibition of Spike–ACE2 interaction has been suggested as an alternative antiviral strategy to prevent and treat COVID-19 disease. Compared with SARS-CoV, the SARS-CoV-2 Spike protein presents two distinctive features that may enhance its affinity to ACE2 and, therefore, its virulence. Indeed, cryogenic electron microscopy has demonstrated that SARS-CoV-2 Spike contains six mutated residues in the receptor binding motifs that increase the affinity for ACE2 and the severity of COVID-19 [10,89,90]. Moreover, SARS-CoV-2—but not SARS-CoV—Spike protein contains an insertion of four aminoacids between S1 and S2 subunits. This insertion constitutes a cleavage site for Furin that facilitates maturation of the Spike protein and, similar to other pathogenic viruses, increases pathogenicity [85,91]. 

ACE2 is a homolog of the angiotensin-converting enzyme that converts angiotensin (Ang) II into Ang 1 and 7, reducing contractile cardiac function [92] and accelerating pathological ventricular hypertrophy upon cardiac stresses [93]. A first pharmacological strategy to reduce SARS-CoV-2 infectivity is blocking or reducing the interaction between Spike viral protein and host ACE2 receptor. One explored strategy, still to be fully proven, is in the utilization of small-molecule inhibitors (SMIs), which lack immunogenic side effects, is easily administered, and seems to be less strain- and mutation-sensitive compared with conventional antibodies [94].

Based on the presence of the more severe symptoms manifested from patients infected by COVID-19 in the presence of pre-existing CVD, probably due to an increased secretion of ACE2, one of the proposed mechanisms of acute myocardial injury caused by SARS-CoV-2 infection and of the increased infectivity of patients with pre-existing CVD has been linked to ACE2 levels. However, conflicting data indicate that the binding of Spike to ACE2 causes ACE2 downregulation, leading to an increase in Ang II and pulmonary vascular permeability, pulmonary oedema, and a compromised lung function. Few in vivo studies based on Ace2 knockout mice treated with recombinant ACE2, or Losartan, produced a phenotype similar to that from humans with CAD, protecting SARS-CoV-2-infected mice from hearth failure and lung injury [32,95,96]. The utilization of engineered human tissues allowed Monteil et al. [97] to observe that clinical grade human recombinant soluble ACE2 reduces SARS-CoV-2 infection. Although their study focused on lungs, the data are promising and suggest an alternative therapeutic intervention in the early stages of viral infection 

Since ACE2 is the receptor for SARS-CoV-2, clinicians were reluctant to use renin-angiotensin inhibitors in patients infected by COVID-19, as this treatment promotes ACE2 expression [98]. Hence, the opportunity to suspend these medications in patients infected by COVID-19 has been debated. Based on randomized clinical trials, several medical societies, including the American Heart Association (AHA), the European Society of Cardiologists (ESC), the Chinese Society of Cardiology, and the Heart Failure Society of America have recommended continuing these therapies, since their use did not affect the susceptibility to SARS-CoV-2 infection or increase the risk of severe illness among patients infected by COVID-19 [27,99,100]. An analysis of the major clinical trials dealing with the utilization of angiotensin receptor blockers (ARBs) suggests that the effect of ACE and ARBs inhibitors in patients infected by COVID-19 is strongly dependent on the severity and stage of the disease, on the time at which COVID-19 is diagnosed, on the dosage administered daily, and on the duration of the treatment [101]. These data could partly explain the discrepancies reported by Dongchao Lu and colleagues, where miR-220c inhibition of the ACE2 transcript could prevent SARS-CoV-2 related inflammatory effects in CVD patients [78].

### 5.3. RNA-Based Therapeutic Strategies

RNA-based drugs, in particular those utilizing ncRNAs, emerged as novel attractive approaches and, therefore, are currently at the stage of clinical trials to treat viral infections or CVD. All miRNAs and lncRNAs reported here have the potential to be targets in developing RNA-based therapeutic strategies against CVD associated with SARS-CoV-2 infection. The studies reported hereafter analyzed miRNAs and lncRNAs function using viral vectors or oligosequences to inhibit or overexpress ncRNAs, chemical inhibitors or agonists, or genetically modified mice. RNA modifications are usually adopted to lower off-target effects and to enhance cellular uptake. Chemical modifications, such as phosphorylation, cap-ending, or polyT/A tailoring are used to increase RNA-oligo stability against RNases.

#### 5.3.1. miRNAs Inhibitors

The currently adopted miRNA inhibitors are small interference RNAs (siRNAs), antago-miRs, locked nucleic acid (LNA) anti-miRs, and Target Site Blockers (TSBs) [102]. SiRNAs are chemically modified RNA duplexes that complementarily bind the loop region of miRNAs. Antago-miRs are oligonucleotides with different types of 3′ conjugations (2′-O-Me, 2′ O-methoxyethyl, 3′ cholesterol, or 2′-fluoro conjugation) to reduce off-target effects and to increase their cellular uptake and binding. Fully complementary to the mature miRNA sequence, antago-miRs contain an additional phosphorothioate backbone linkage, in which sulfur replaces one of the nonbridging oxygen atoms in the phosphate group [103]. LNA-anti-miRs comprise a class of bicyclic RNA analogues in which the furanose ring in the sugar-phosphate backbone is chemically locked in an RNA mimicking N-type (C3′-endo) conformation by the introduction of a 2′-O,4′-C methylene bridge. LNAs increase the efficiency of anti-miRs compared with antago-miRs. MiRNAs TSBs are LNA-enchanced antisense oligonucleotides designed to bind a specific miRNA binding site of an mRNA or lncRNA, thereby competing with miRNA access to that site. It has been proven that TSBs selectively inhibit a certain mRNA/lncRNA target without affecting the binding of the miRNA to other targets. This further increases cell specificity and target selectivity. 

To avoid any off-target effect and toxicity as well as to increase the efficiency of RNA-based drugs, most of the current RNA therapies are administered via systemic intravenous, anterograde, or retrograde infusion (i.e., miR-92a). Catheter-based delivery of anti-miRs to the heart did not show significant benefits in terms of systemic miRNA inhibition. Alternatively, drug-eluting stents or balloons have been tested with some promising results to deliver anti-miRs, such as miR-21 and miR-126-3p.

#### 5.3.2. LncRNA Inhibitors

MiRNA and lncRNA TSBs are LNA-enhanced antisense oligonucleotides designed to bind a specific miRNA binding site of an mRNA or lncRNA, thereby competing with miRNA access to that site. It has been proven that TSBs selectively inhibit a certain mRNA/lncRNA target without affecting the binding of the miRNA to other targets. This further increases cell specificity and target selectivity. 

LncRNA function is usually inhibited using antisense oligonucleotides (ASOs), siRNAs, and LNA-Gapmers [102]. Several lncRNAs play functional roles in the nucleus. Hence, since ASOs and siRNAs preferentially inhibit lncRNAs in the cytoplasm, LNA-Gapmers are widely used to efficiently inhibit nuclear lncRNAs by inducing ribonuclease H-dependent cleavage of the lncRNA target. 

#### 5.3.3. miRNAs and lncRNAs Inhibitors of Clinical Relevance

MiRNAs became the first candidate among all ncRNAs to be used in the design of RNA-based therapies, since they are highly conserved and stable and exhibit very short sequences. Although miRNA-based therapies reached clinical trials, data are still poor compared with strategies using antisense oligosequences against mRNAs. Among miRNA-based therapies in clinical trials, the miR-122 antagonist Miravirsen has been shown to reduce HCV infection [47]. However, it did not proceed beyond phase I. MiR-21 is promoted upon DENV infection [44] and is reported to contribute to myocardial diseases by stimulating MAP kinase signaling in fibroblasts [75]. As profibrotic miRNA, the first clinical trials focused on the generation of antago-mirs (RG012) against miR-21, with positive effects against fibrotic kidney disease. The RG012 anrimiR-21 is currently in a phase II trial with promising preliminary efficacy in reducing renal dysfunction [75]. A phase I trial started for MRX34, a miR-34a mimic that shows potential immunogenicity and selectivity, with off-target effects [51]. Remlarsen is a miR-29a mimic that has been utilized to reduce collagen expression in multiple fibrotic conditions, including the heart, the liver, and the lungs. Since miR-29a promotes heart remodeling after MI, Remlarsen successfully reached phases I and II [104]. A phase I trial started for MRG-110, a miR-92a antago-miR adopted against ischemic heart conditions to promote angiogenesis and neovascularization after MI [105]. A single injection of encapsulated MRG-110 in microspheres promoted heart remodeling and was also beneficial against athero-progression in mice and rats [105]. 

Since lncRNAs are poorly conserved between species, only those with high locus-conserved transcripts have been considered for new therapies. Indeed, despite sequences potentially diverging, a conserved genomic location may reflect a similar or conserved function [106]. Among those lncRNAs reported by Wucher and others highly conserved in at least seven divergent species emerged NEAT1, MALAT1, H19, CHROME, and HOTAIR [107,108,109]. 

#### 5.3.4. Viral Vectors and Current RNA-Based Vaccines

LncRNA and miRNA overexpression is achieved only using viral vectors, which show an increased cell-type specificity and efficiency compared with all adopted mechanisms. Adeno-associated viruses (AAV) have been successfully reported to improve the therapeutic cardiac delivery of miR-1 and miR-199a [102]. However, the efficiency reported for AAV-mediated gene therapy in recent human trials indicated that AAV-associated RNA delivery must be significantly improved [110].

Recent findings indicates that to favor the LNA-based oligosequence administration in humans, to improve their efficiency, and to avoid off-target effects, the physicochemical and biological stability of RNA-based drugs must be implemented. Accordingly, current mRNA-based vaccines against SARS-CoV-2 infection have been modified or combined with deoxyribonucleotides within the RNA sequence to create more stable and efficient LNA-RNA mixmers. Moreover, to increase their availability in humans, RNA-based sequences have been encapsulated into liposomes and delivered as lipid nanoparticles [111]. 

At the moment, all nine SARS-CoV-2 authorized vaccines are classified as inactivated vaccine and viral vector vaccines [112]. Sinopharm and Sinovac are two inactivated vaccines derived from viruses grown in culture and then chemically inactivated [112]. Among these vaccines delivering the Spike protein as a full-length recombinant protein subunit to host cells, there is only Novavax (US), with an 89% efficacy against COVID-19. Replication-incompetent AAVs have been used for several vaccines against HIV, Ebola, and malaria viruses. The same approach was used to deliver the full-length, stabilized SARS-CoV-2 Spike protein by the AstraZeneca and Johnson & Johnson vaccines [112]. 

As described for miRNA- and lncRNA-based oligos, lipid nanoparticles improved vaccine administration. They were used to protect the perfusion-stabilized Spike mRNA alongside the entrance in the host cell by the Pfizer-BioNTech and Moderna vaccines, demonstrating a more than 90% efficacy against SARS-CoV-2 infection and clinical disease. However, current vaccines show a reduced efficacy against new SARS-CoV-2 variants. Linked to CVD, so far, a two-dose regiment of the ChAdOx1 nCoV-19 (AstraZeneca) vaccine did not show protection against mild-to-moderate COVID-19 due to the B.1.351 variant [113].

## 6. The 5′ Leader Sequence of SARS-CoV-2 as Target for ncRNA-Based Therapies

Small RNA interference (siRNA) technology has been already approved to efficiently inhibit viral infections in host cells, since siRNAs can selectively inhibit the transcription of viral proteins, such as the poliovirus and Rous sarcoma viruses [114,115]. Similarly, synthetic vector-derived siRNAs were efficiently used in vitro and in mice to inhibit hepatitis B and C virus by targeting critical genes responsible for viral replication [116,117].

RNA interference is a native, gene-specific process conserved in almost all cells and organisms. It was therefore considered an appropriate mechanism to attenuate coronavirus viral infection and replication in mammal cells and, therefore, a promising therapeutic approach for the treatment of COVID-19 disease [118].

The in vitro study by Lu and collaborators indicated that treatment of African green monkey kidney Vera-E6 and HeLa cells expressing an RNA-dependent RNA polymerase (RDRP) of SARS coronavirus with short hairpin RNAs (shRNAs) efficiently inhibits the expression of RDRP [118]. Lu and coworkers also reported that shRNAs can be easily incorporated into viral vectors for in vivo delivery, supporting the candidate efficiency of an iRNA-based therapeutic approach. 

Similarly, other studies have demonstrated that siRNAs designed to selectively target and inhibit Spike mRNA transcript could effectively and specifically inhibit Spike protein in SARS-CoV-infected monkey kidney cells and in the human embryonic kidney 293T cell line [119,120]. Some of the most promising results have been presented by Li and coworkers, elegantly demonstrating that specific siRNA duplexes targeting the SARS-CoV Spike and ORF1b regions can significantly suppress SARS symptoms in infected macaques [54]. However, since these studies were conducted on the SARS-CoV virus and focused on specific genes of the viral genome, such as the Spike protein, they did not take into consideration the high mutation rate of viral genome and, therefore, a possible reduced efficacy of siRNA targeting. 

Current coronavirus genomic comparisons, in silico predictions, and first in vitro studies all started from the hypothesis of directing RNA-based therapies against highly conserved and poorly modified sequences in the SARS-CoV-2 genome to successfully inhibit viral replication. In this perspective, Zeng and collaborators first reported that coronaviruses contain a highly conserved leader sequence of 63 bp and an additional intergenic sequence (IGS) of 9 bp located at the 5′UTR of the genomic RNA, fused to all mRNA body sequences [121]. Although their function is not completely known, the leader sequence and the IGS sequences, later referred to as cis-acting transcription-regulating sequences (TRSs), are crucial for proper gene expression during viral replication [122,123]. A putatively conserved TRS sequence TAAACGAAAC has also been identified through manual alignment that is upstream from the leader sequence and conserved among SARS-CoV and SARS-CoV-2, indicating a similar replication process to those of other coronaviruses [123,124,125,126]. Genome sequence comparison identified a conserved CUAAAC consensus in the leader sequence in all SARS and coronavirus genomes. Both TRSs and the leader sequence are needed to guarantee efficient accumulation of SARS-CoV-2 mRNA transcripts and proteins during infection since they protect viral mRNAs from endonucleolytic cleavage of the mRNAs cap [123,124,125,126].

A number of in silico studies analyzed the common features of viral 5′UTR, identified conserved motifs in the TRSs and the leader sequence selectively recognized by miRNAs, and identified specific lncRNAs that may negatively modulate viral genome expression and possibly reduce SARS-CoV-2 infectivity [53,56,73,127]. Baldassarre and coworkers [127] described a putative binding of miR-4507, miR-638, miR-3150b-3p, and miR-602 highly expressed in the lung at the 5′UTR of SARS-CoV-2. Using a meta-analysis approach, Mohammadi-Dehcheshmeh and collaborators [53] identified miR-5004-3p as unique human miRNA binding the leader sequence. 

Our laboratories have also identified several miRNAs binding the leader sequence of SARS-CoV-2. In particular, only a number of miRNAs containing a GGG nt triplet in their seed sequence were identified as able to selectively bind the “AACnAAC”, “AUACCUUCCA”, and “nUnGAUCUnU” motifs within the leader viral sequence [56]. Among the identified miRNAs, several are known modulators of viral infection and related inflammation, such as human miR-3150b, miR-4531, miR-3661, miR-3144, miR-30c-1, and let-7c. Moreover, miR-3661 is involved in SARS-CoV-2 protein synthesis [2,52]. Other miRNAs are also involved in cardiovascular diseases, supporting their role as candidates in developing RNA-base drugs against SARS-CoV-2 infection and related cardiovascular complications.

A hypothetical therapeutic strategy using RNA oligos against the leader sequence has been proposed by Kim and colleagues [128], who identified a translation initiation site (TIS) located in the leader sequence, a non-canonical CUG codon that is likely to act as a master regulator of SARS-CoV-2 translation. Blocking this region with antisense oligonucleotides disrupts TIS-mediated translation of SARS-CoV-2 [129]. Additionally, studies by Li and collaborators indicated that treatment of SARS-CoV-infected Vero E6 cells with siRNAs against the leader sequence inhibits gene expression and viral replication in cultured cells [54] and that the efficiency of siRNAs was higher compared with antisense oligonucleotides. 

The data reported by Moazzam-Jazi and coworkers indicate that several human lncRNAs preferentially interact with the 5′ end of SARS-CoV-2 rather than the 3′ end [66], reinforcing the possibility to design RNA-based vaccines against the 5′ end of the virus. Accordingly, H19, MIAT, FENDRR, and linc01505 show BS against the SARS-CoV-2 leader sequence [53,56,73,127].

Even though current findings indicate that the leader sequence may represent a promising site against which drug-based and RNA-based therapeutic interventions should focus on, experimental validations of miRNA interaction with SARS-CoV-2 5′UTR are still missing. The combination of in silico predictions and interactions, ex vivo studies, in vitro experiments, and large animal models should be considered a crucial starting point to select promising ncRNA candidates [6].

## 7. Conclusions

The very high transmission rate of COVID-19 worldwide in early 2019 forced the WHO to declare it a pandemic in 2020. In addition to respiratory disorders, several patients infected with SARS-CoV-2 developed severe pathologies involving the cardiovascular system. Similarly, patients with pre-existing CVD showed a dramatic increase in the release of inflammatory cytokines and an increased risk of inflammatory exacerbation and mortality. The combination of these two observations clearly suggests a functional interaction between SARS-CoV-2 infection and the cardiovascular system. The overwhelming need to treat patients infected by COVID-19 and the unavailability of selective therapies against SARS-CoV-2 has led physicians and scientists to adopt clinically approved antiviral and anti-inflammatory drugs to treat patients infected by COVID-19. As consequence of this emergency, it was not possible to verify if and, possibly, to which extent these therapies were associated with adverse effects, an event inevitably encountered, especially in patients with CVD showing undesirable pharmacokinetic interactions. Moreover, most of the data published in a relatively short time often refer to a small number of patients, if not even to a single clinical case. For this reason, data based on a larger number of patients are dramatically needed to draw appropriate (almost) final conclusions regarding possible interactions between currently adopted antiviral therapies and drugs in use for an effective treatment of patients with CVD.

In addition to currently used therapies, at the moment, nine SARS-CoV-2 authorized vaccines are in use and classified as inactivated vaccines and viral vector vaccines, used to induce the expression of the viral protein Spike within host cells and to generate a specific *anti-Spike* antibodies-mediated immune response. Although the efficacy of RNA-based novel vaccines is higher than that of previous vaccines, the high mutation rate reported in the Spike protein suggests that therapies based on Spike protein may be ineffective in a long-term perspective. Moreover, unlike many conventional vaccines, the use of Spike to activate the host antiviral response would inevitably require the constant production of new vaccines able to protect against new SARS-CoV-2 variants. This procedure could not be favorable in terms of cost–benefits and would lead patients with cardiovascular problems to undergo vaccination cycles not devoid of possible side effects. Are we therefore considering the wrong target in designing RNA-based vaccines?

## 8. Future Perspectives

**Figure 1:**
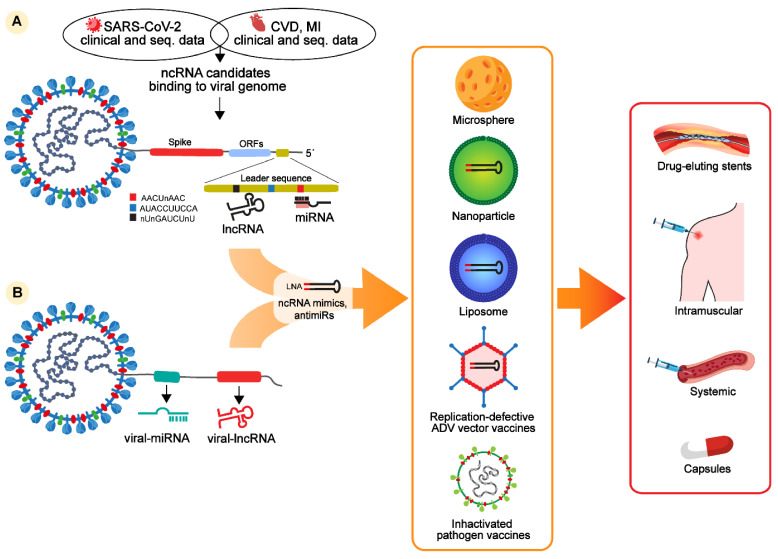
Host and viral noncoding RNAs and potential therapeutic strategies to treat COVID-19 patients with cardiovascular complications. Noncoding RNA-based therapies derived from (**A**) clinical and sequencing data from patients with COVID-19, CVD, or MI and from patients with cardiac and COVID-19 diseases indicate that several ncRNAs, such as miRNAs, lncRNAs, and circRNAs, are differentially regulated in these patients and contain putative binding sites in the Spike transcript or in the leader sequence of the SARS-CoV-2 genome. Binding specificity might be enhanced for miRNAs binding three motifs conserved in the leader sequence of the SARS-CoV-2 genome, named AACUnAAC, AUACCUUCCA, and nUnGAUCUnU. Potential ncRNAs interacting with the leader sequence can be used to design LNA-oligos mimicking (LNA-RNA mimics) or inhibiting (LNA-anti-miRs) selected ncRNA candidates. (**B**) Alternatively, recent findings identified the presence of miRNAs and lncRNAs as alternative candidates to design RNA-based inhibitors against viral infection. NcRNA-based mimics or anti-miRs can be encapsulated in microsphere, lipoparticles, or inactivated viral vectors as vaccines to be systemically or intramuscularly delivered. Certain ncRNAs are already in phases I and II of clinical trials as drug-eluting stents. Alternative administrations are in clinical trials, such as modified and stabilized ncRNAs delivered as capsules.

RNA-based drugs are currently at the stage of clinical trials for the treatment of viral infections or CVD. Those based on ncRNAs, such as miRNAs and lncRNAs, seem to be capable of targeting both the Spike protein transcript and low mutation regions of the SARS-CoV-2 genome. Indeed, growing evidence supports the existence of potentially non-mutagenic regions located at the 5′UTR of the viral genome, including the leader sequence, containing specific motifs for selective ncRNA binding. Hence, it seems definitively plausible to consider this region as a starting point for the design of new RNA-based drugs to treat patients infected by COVID-19, including those with CVD. The targeting of ncRNA-based drugs against noncoding viral regions has the potential to lower the rate of adverse effects and undesirable pharmacokinetic interactions (Figure 1).

We can also hypothesize the possibility of exploring an opposite avenue: the discovery of the existence of viral miRNAs and lncRNAs, distinct from host ncRNAs, opens up the possibility to utilize them as targets for novel specific drugs as more selective and effective therapies against SARS-CoV-2 infection (Figure 1).

Even though the COVID-19 pandemic seems to be somehow under control, new viral variants are evolving, continuously placing the entire world population at risk, including people who already received a complete cycle of vaccines based on the original SARS-CoV-2 strain, which are therefore relatively non-effective against new variants. There is still the need to develop efficient pharmacological tools for the treatment of infection by taking into account the equally dramatic associated consequences, such as those affecting the cardiovascular system.

The lessons learned facing SARS-CoV-2 could be pivotal in the future in the unwanted but unfortunately probable event that other spillover pathologies arise, seriously threatening humans. COVID-19 was not the first, and most probably will not be the last.
